# An assessment of the potential for the development of the shale gas industry in countries outside of North America

**DOI:** 10.1016/j.heliyon.2018.e00516

**Published:** 2018-02-07

**Authors:** Minh-Thong Le

**Affiliations:** a GAEL, CNRS, Grenoble INP, INRA, Univ. Grenoble-Alpes, 38000, Grenoble, France; b Faculty of Economics and Business Administration, Hanoi University of Mining and Geology, Viet Nam

**Keywords:** Economics, Energy

## Abstract

The revolution of shale gas in the United States (the US) has become a phenomenon at the beginning of the 21st century. It has been significantly influencing the United States’ economy and the global gas market. Like America, other countries have also been searching for shale gas. However, the conditions for developing this resource are very different among regions and nations. On the other hand, there are also many doubts, debates and even strong oppositions to the development of shale gas because of the complicated issues that arise regarding its extraction, and also due to the fact that its impacts are not fully known. Therefore, at present, the development of shale gas is still a big question for regions, countries that have potential and desires to exploit such resources. Although it is difficult to identify all necessary or sufficient conditions to develop shale gas, the experiences of the United States could be instructive for other countries. In this article, the potential development of shale gas in China and Europe is analyzed, which relies on the fundamental conditions considered as important factors for the success of the shale gas industry in the US. Through these analyses and we demonstrate the difficulty of developing this resource outside North America.

## 1 Introduction

In the early 2000s, due to a decrease in the production of conventional gas in the United States, as well as the growth of natural gas demand in many sectors, the American government took strong actions on research and development of unconventional gas resources, particularly shale gas. Thanks to many favorable conditions such as technological innovations, favorable policies, free market, developed infrastructure and the advantages of geological conditions, a boom in shale gas was observed in the US. In recent years, the United States has obtained many benefits from the development of shale gas, which include greater energy security, reduction of unemployment, greater industrial competitiveness on a global scale, and a wide range of other environmental and economic benefits.

**Fig. 1:**
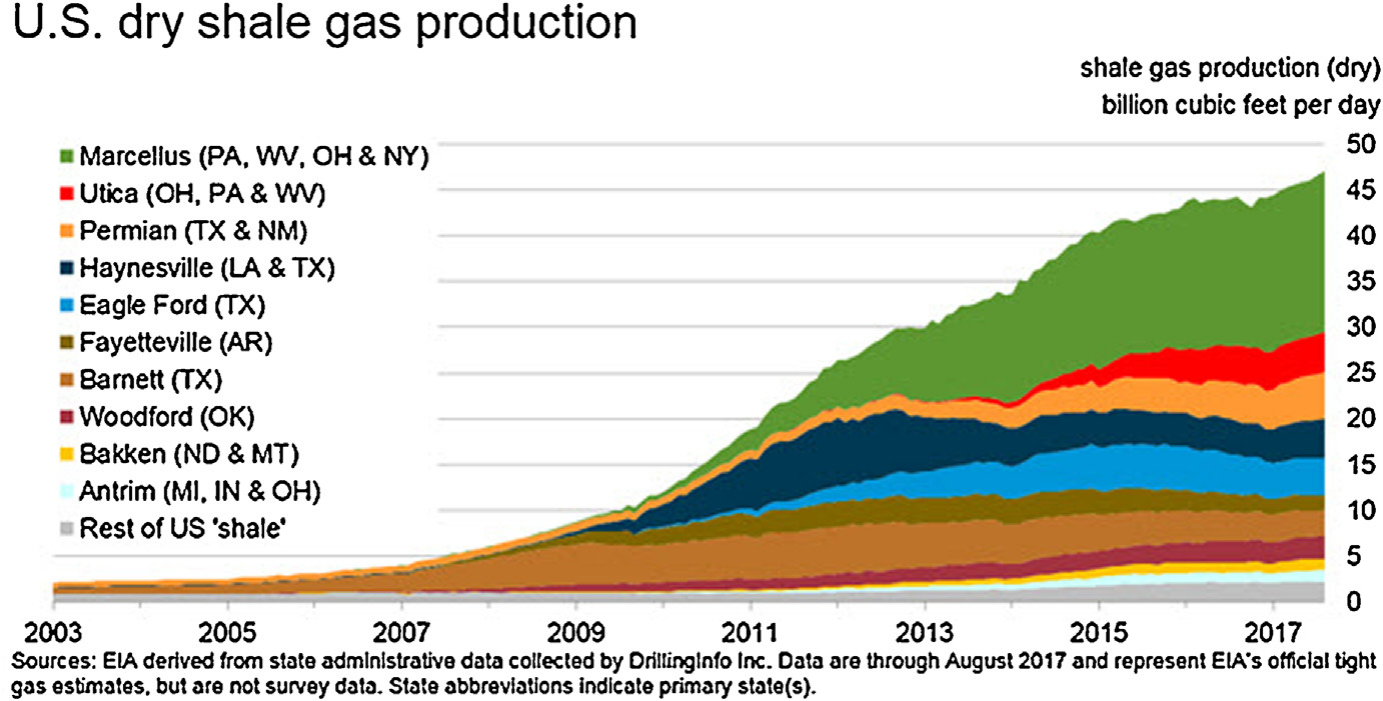
The production of shale gas in the US. Source: EIA (2016).

**Fig. 2:**
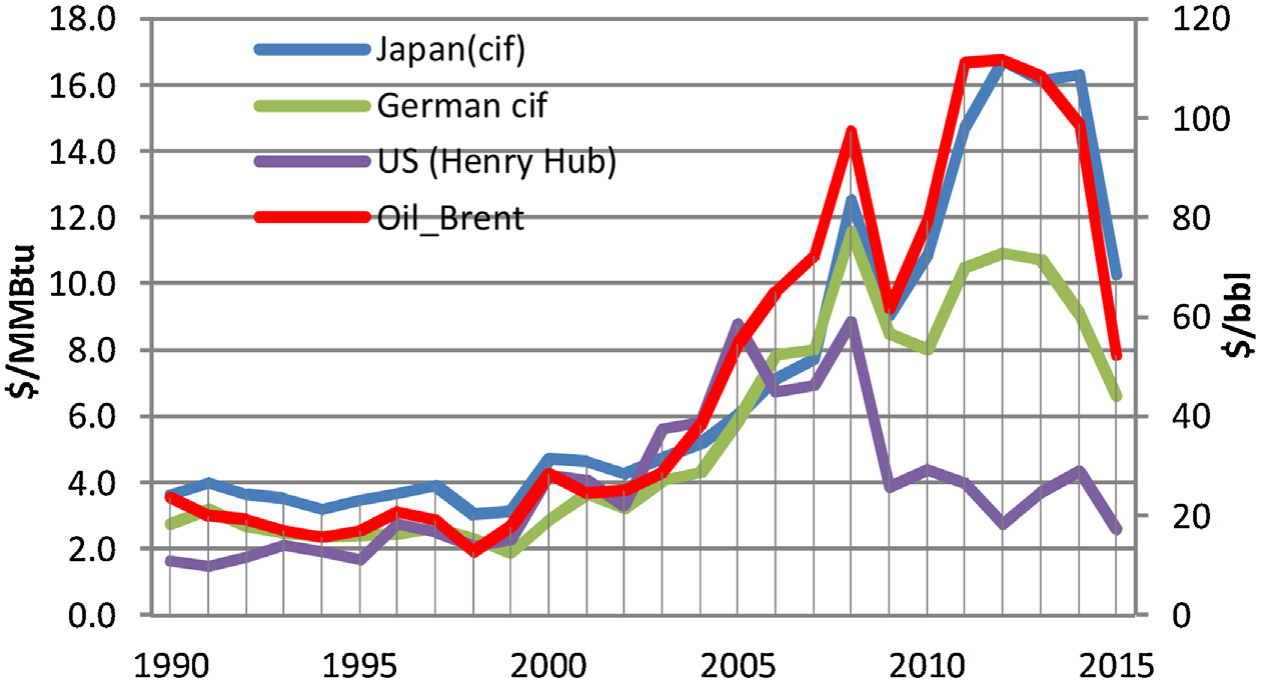
The price of natural gas in the world. Data Source: BP Statistical Review of world energy (2016).

Firstly, thanks to the development of shale gas, the reserves of natural gas in the US rose sharply. Extraction of shale gas has not only offset the decline of conventional gas output, but also resulted in the growth of total natural gas production. Since 2005, production of shale gas has increased rapidly in the country (see Fig. 1) and it has helped the US to overtake Russia to become the largest gas producer in the world since 2009 (Bros, 2012; Wang and Hefley, 2016). The high availability of unconventional gas, especially shale gas, allows the United States to become a net exporter of natural gas in the world. The revolution of shale gas has brought in a decrease in natural gas price in the United States, from 15 USD/Mbtu in 2008 to about 4 USD in 2014, which is twice to three times lower than that in Europe and four to six times lower than that in Asia (see Fig. 2). This has created many benefits to customers in the natural gas market in this North American country and a new position of the United States in the international energy market.

Secondly, shale gas revolution has already led to economic advantages in terms of states and localities to individual sectors and also to the nation. It has significant impacts on the economic growth of the United States. These economic impacts comprise indirect impacts and induced impacts. The indirect impacts are additional economic activity of the value chain network caused by the economic activity of the direct industry. The induced impacts are additional economic activity of all other unrelated firms and households caused by the economic activity of the direct impacts and the indirect impacts (Hefley and Wang, 2015). It has significant impacts on the economic growth of the United States. The macroeconomic impact of the shale gas revolution is between 0.3% and 1% of the US GDP (International Monetary Fund, 2013). It created a lot of direct and indirect jobs. A report indicates that the shale gas industry supported more than 600.000 jobs in 2013, and this figure would reach over 1.6 million by 2035 (Wang et al., 2014; Hefley and Wang, 2015). The development of shale gas in the United States has been the catalyst for the recovery of traditional industries, such as petrochemical industry, fertilizer producers, plastics and other industries that consume a great deal of energy like aluminum smelters, steel mills and refineries.

Finally, the considerable change of shale gas has contributed to improving the environment. The rapid growth of shale gas production and the significant decline in natural gas price in the United States have led to a reduction in coal consumption in the electricity sector, and an increase in natural gas use in this sector at the same time. For example, in 2012, the production of electricity from natural gas increased from 25% to 30% of the total, while electricity from coal continued to decrease from 42% to 37% of the total production of electricity (Wang et al., 2014). Both reports from the International Energy Agency (IEA) and the Energy Information Administration (EIA) in 2013 showed that carbon emissions from fossil-fuel combustion in the US decreased sharply in recent years. From 2007 to 2012, a six-year period, the United States reduced their emissions of carbon dioxide (CO_2_) of 450 million tons in total – the largest decrease recorded on the whole planet. In 2012, the reduction reached about 70% of CO_2_ emissions set in the framework of the Kyoto Protocol (EIA, 2013a). Carbon emission from fossil-fuel combustion in the US is most likely to be back to the 1990 levels (Wang et al., 2014).

The success of the development of shale gas in the US has created a lot of interest and attention to other countries in the world, in particular, the countries which possess the potential of these resources. According to Speight (2013), there are two groups of countries that emerge with shale gas development. The first group consists of countries that are highly dependent upon natural gas imports. They want to develop shale gas to alter their future gas balance, guarantee their energy security, and reduce the dependence on the importation of gas in general. China and European countries, like Poland, are typical examples of nations having motives for developing the shale gas industry. The second group includes countries where the estimation of shale gas resource is large and there already exists significant natural gas production infrastructure for internal use or for export such as the United States and Canada.

Countries where there is potential for shale gas mostly belong to the first group. They are commonly highly dependent on gas import and the strong growth of natural gas demand, especially the countries in Europe and Asia. However, unlike the reliable database in the United States, there are only a few available estimations of recoverable resources of shale gas in Europe, Asia and other continents. At present, the opinions and guidance on the development of shale gas in the world are exceedingly different. A few countries have exploited this resource such as the United States and Canada. Other countries are developing the shale gas industry such as China, Poland, and England; while many nations like France and Bulgaria disapproved its further development. It has been argued that shale gas development is still a subject of controversy because of many reasons including the uncertainty of resource estimation, or relevant environmental issues such as the pollution of groundwater and surface water, as well as the generation of greenhouse gases. These problems will be presented in more details in the Section 2.2.

The purpose of this paper is to analyze and assess the potential development of shale gas outside the United States in current conditions. The analysis will focus on the Europe and China – the two areas having strong dynamics in the development of shale gas after the success of the US. Moreover, the analysis is mainly based on the factors and fundamental conditions that led to shale gas revolution in the US in recent years. The assessment takes into account current changes such as the drop of oil prices that has an impact on the gas market, the variations in energy policy and climate policy of regions and countries. Analysts also indicate the difficulties and challenges that involved nations will have to face in the process of shale gas development.

## 2 Background

### 2.1 The potential and prospect of shale gas in the world

In the present context, the problem of climate change has been creating a lot of challenges for humanity. This urgent issue has a strong influence in the energy policy of countries. The demand for clean energy sources increases significantly and has become one of the factors that lead to the urgent issue in energy security in many nations, as renewable energy sources have not yet replaced traditional ones such as coal and petroleum. Natural gas is regarded as an important energy source in the structure of energy transition in hopes of hindering climate change. All signs indicate that the future gas demand will increase and hold a progressively more important position in the global primary energy mixture. According to the World Energy Outlook 2013 of the International Energy Agency, the demand for gas is expected to rise to 48% by 2035 (IEA, 2013). It is especially true with the countries that need much energy to develop its economy like China, or the countries that depend strongly on the gas import such as European countries.

The IEA anticipates that China will consume more gas than the European Union in 2035 (IEA, 2011). As a consequence of the revolution of shale gas in the US, we have witnessed an explosion of interest in shale gas in China. In the future, shale gas will be an important energy source, which would help to ensure energy security, and gas demand in China will increase substantially. China is in various stages of evaluation of its shale gas resources and has ambitious goals for the future production. If these targets are achieved, the impact on the regional gas markets and on the international market will be very strong (Andrews-Speed and Len, 2014).

In Europe, although government support ensures alternative energy sources, such as renewable energy, that will represent a larger percentage of the total energy supply; according to the judgments of experts, the hydrocarbons are widely expected to dominate the European energy mixture at least to 2030 (Gracceva, 2012). The secure supplies in the future become an important issue in the energy policy of the European countries, and the development of new indigenous gas resources, particularly shale gas, is an attractive proposition (Aurélien Saussay, 2014). Shale gas may help the EU to cope with its future of energy demand, as well as to reduce the dependence on imported gas by increasing indigenous production levels.

Geologists have long known about the existence of shale formations, but technical and commercial access to those resources is new. Although, the knowledge level regarding the amount of shale gas that is economically recoverable has rapidly increased over the last decade, until now many aspects concerning shale gas are still unknown or uncertain. There have been no reliable estimates of shale gas resources outside the United States. It is therefore difficult to determine the relative magnitude of shale gas in undiscovered shale plays in the world in comparison to those in known shale plays (McGlade et al., 2012). The exact size of the base of shale gas resources varies depending on sources of information (Medlock, 2012). Table 1 below analyses the most recent estimates of three major reports on resources of shale gas in place and shale gas technically recoverable in the world.

**Table 1:** Estimates of shale gas resources in the world.

Continent	Shale gas in place			Shale gas technically recoverable resources	
	Rogner (1997) (Tcf)	EIA (2011) (Tcf)	EIA (2013a,b) (Tcf)	EIA (2011) (Tcf)	EIA (2013a,b) (Tcf)
North America	3842	7140	9291	1931	1685
South America	2117	4569	6390	1225	1431
Europe	549	2587	4895	639	883
Africa	1548	3962	6664	1042	1361
Asia	3528	5661	6495	1389	1403
Australia	2313	1381	2046	396	437
Others	2215	Na	na	na	na
Total	16,112	25300	35782	6622	7299

The estimation of shale gas resources in the world changes over time. At present, according to the study of EIA, the technical recoverable shale gas resources are concentrated in the United States (North America); Poland, France, Norway (Europe); China (Asia); South Africa, Algeria (Africa); Argentina, Brazil (South America); and Australia. Hence, there are multiple substantial uncertainties about assessing the recoverable volumes of shale gas, on both a regional and a global scale even in regions where gas production is currently taking place, notably North America. A significant uncertainty about the actual size of the resource at the place still remains, creating considerable variations in the available estimates (McGlade et al., 2013a, McGlade et al., 2013b).

The uncertainty of the shale gas resource estimates will have a powerful impact on the future of the shale gas industry and national energy policies. The potential for cost-effective production of shale gas is still unknown. The structural differences between the potential production areas such as transport infrastructure, thermal power stations operating on gas availability, or the presence of oil services industry that control the exploitation of unconventional resources complicate the definition of economically recoverable resources (Pearson et al., 2012).

### 2.2 The challenges of shale gas development

“The revolution” of shale gas in the United States has achieved a lot of success. However, the development of shale gas is facing numerous obstacles and challenges. By analyzing the development of shale gas in the United States, we indicate the fundamental challenges that countries hoping to develop shale gas have to face.

#### 2.2.1 The water demand

The production of shale gas consumes a large volume of fresh water. On average, we need around 12–20 million liters of water per horizontal well in shale gas production. The amount of essential water for the hydraulic fracturing process depends on the type of shale gas and the fracturing operations. The water consumption will grow as the numbers of wells and shale gas products increase. Certainly, such a large volume of water, and a high rate of withdrawals from local surface or ground water sources would have a significant impact on the local water system. The consumption of water is particularly important in areas where dry weather conditions often strictly limit the availability of water and its use (Richardson et al., 2013). Consequently, the development of shale gas poses a big challenge in regions or countries where there is a lack of water.

##### 2.2.1.1 The capacity of pollution of the groundwater and surface water

The chemicals comprise between 0.5% and 2% of hydraulic fracturing liquids, many of them are toxic and carcinogenic (Wang et al., 2014). These fluids are injected directly into the ground and they can affect groundwater sources. In addition, the flow-back or “produced” water from fracturing fluids might contaminate the water surface. They may adversely affect human health and the environmental quality if they are untreated or directly discharged onto the land or into streams, rivers and lakes.

##### 2.2.1.2 Generation of greenhouse gases

Shale gas contains more than 90% of methane (CH_4_), which may contaminate the air and the water. In particular, methane is a very powerful greenhouse gas compared to carbon dioxide. The effects of shale gas on climate change have become more complex to evaluate and controversial, partly because of the uncertainty about the scale of methane leaks. Some researchers worry that expanded production of shale gas could result in the increase in the release of methane as fugitive emissions during the drilling, completion, production, transportation and use of natural gas. Thus the fugitive emissions in the process of shale gas development would probably lead to a net increase in green-house gas emissions.

##### 2.2.1.3 The price of natural gas

Shale gas is profitable if the price of natural gas is offset against the production cost. The real marginal cost of production in the US reached $4–$6 per MMBtu (EIA, 2013a), while estimates of shale gas production costs in other regions are higher. In Europe break-even costs range from roughly 8 to 12 USD/MMBtu (Gény, 2010), in China this number is higher with about $12 per MMBtu. However, the production cost of shale gas may decrease thanks to technology progress, but the current price of gas in the international gas market is too low compared with the production cost of shale gas (see Table 2). Recently, according to the data of the International gas report in April 2016, the price of gas at Henry Hub in the United States is very low (about 2$/MMBtu); in the Japanese gas market, LNG prices reach nearly 4.5 USD/MMBtu; in the European market, the natural gas price at National Balancing Point (NBP) is about 4 USD/MMBtu (Platts, 2016). In comparison with the price at the end of 2014, price of gas in the Europe and Asia market dropped approximately about 3 times.

**Table 2:** Comparison gas price and production cost of gas.

	US (Henry Hub)	Europe (NBP)	Asia (JKM – Japan)
Price in the period of 2005–2014 (USD/MMBtu)	2.76–8.85	4.85–11.56	6.05–16.75
Price in 2015 (USD/MMBtu)	1.8–3.5	5.81–9.5	8.79–15.5
Estimation of production cost (USD/MMBtu)	4–6	8–12	>12

As consequence of the falling of crude oil price in recent years, the price of natural gas in the international gas market has also been decreasing dramatically. This price is lower than the marginal cost of shale gas production in a long term. Therefore, with the price too low at present, it has reduced the interest of investors in shale gas. In fact, there are a lot of gas producers who have been reducing their production scale as well as abandoned their investments in projects of shale gas in the United States and other regions in the world. Even some companies were on the verge of bankruptcy.

On the contrary, when the natural gas price is high, it attracts more investments in this sector. However, in the sector of shale gas, investors must spend a large amount of money than in conventional gas projects. Moreover, the life cycle of well shale gas is shorter than that of the well conventional natural gas, and the production of shale gas declines rapidly after the peak of production. Hence, it is necessary to continue the supply of capital investment to maintain the production. In addition, if gas price is high, customers in the electricity sector will look for cheaper energy sources like coal to replace gas. As a result, the investment in shale gas has a high risk level, because of both uncertainty and difficulty.

##### 2.2.1.4 The opposition from the population

A very important issue of the development of shale gas is whether people accept the activities related to its production or not. According to EIA, in the publication “Golden rules for a golden age of gas” in 2012, the necessary to build a “social license to operate” was emphasized (IEA, 2012). Therefore, the absence of social acceptability, even the hostility of the majority of the population towards the development of shale gas will be a great restriction in the future, in particular in European countries.

##### 2.2.1.5 The uncertainty of resource estimation

The uncertainty of the estimates will significantly influence the future of shale gas industry and national energy policies. Therefore, the potential profitability of shale gas is still difficult to predict. Except the United States, other countries having significant potential for shale gas development have a shortage of reliable estimates on the shale gas resources. For that reason, these resources are still unclear and it is a great obstacle for countries which desire to develop their shale gas.

For example, the estimate of the EIA indicates that China has the largest shale gas reserves in the world, almost twice as large as those of the United States. These reserves represent almost 20% of the reserves in the world, and about 92% in Asia. Shale gas reserves in China contain roughly 13 times more gas than its conventional gas resources remaining to be recovered (Davies and Tomsen, 2013). However, there is no consensus in the value of shale gas resources in China. Quantity estimates of shale gas resources vary greatly. Most estimates are from fundamental institutions outside China. The main method in China's shale gas estimations has been used including comparison and analogy, so the accuracy of the results is little and uncertain (see Table 3). The drilled holes in China have been very limited, thus the detailed geological information on shale formations is inadequate and unavailable.

**Table 3:** Estimates of the potential of shale gas resources in China.

Evaluating Institution	Technically recoverable reserves (Tcm)			Year
China University of Geosciences	26			2008
Research Institute of Petroleum Exploration and Development, Petro China	10–20			2009
China National Petroleum Corporation – CNPC	21,5–45			2010
The US Energy Information Administration – EIA	36,1			2011
Center for of Oil and Gas Strategic Research, Ministry of Land and Resources of China	25,1			2011
International Energy Agency – IEA	26			2012
The US Energy Information Administration – EIA	31,5			2013
Christophe McGlade et al.	Low	Average	High	2012
	4,2	19,2	39,8	

The shale gas industry in Europe is in its infancy. European countries were reported to possess about 10% of world reserves of shale gas (Pearson et al., 2012). However, the continent has little knowledge about the potential, quality, precise locations of sweet spots of its shale gas resources. Only a small number of exploration wells have been drilled in Europe. In the absence of actual exploration and production data, any translation of technically to economically recoverable reserves is fraught with significant uncertainties. Similar to China, there is so much uncertainty with regard to European shale gas resources. The high uncertainty surrounding the estimates for technically recoverable reserves of Europe is presented in Table 4. There is a general tendency to reduce the estimates of technically recoverable resources. For example, according to the EIA, Poland had been extremely optimistic about the prospects of shale gas. Nevertheless, the Polish Geological Institute has reduced the estimates of technically recoverable resources from 5300 Bcm to 346 – 768 Bcm – a reduction to about one-tenth of the original estimates (Polish Geological Institute, 2012).

**Table 4:** Estimates of the potential of shale gas resources in Europe.

Source	Country/region	In place (Tcm)		
Rogner (1997)	Western Europe	14.4		
	Central and Eastern Europe	1.1		
		Technically recoverable Resources (Tcm)		
Wood Mackenzie (2009)	Europe	4.2–5.6		
CERA (2009)	Europe	3–12		
EIA (2011)	Europe	18.1		
McGlade et al. (2012)	Europe	15.9		
EIA (2013a,b)	Europe	16.9		
Pearson et al. (2012)		Lowest	Mean	Highest
		2.3	8.9	17.6

## 3 Analysis

**Fig. 3:**
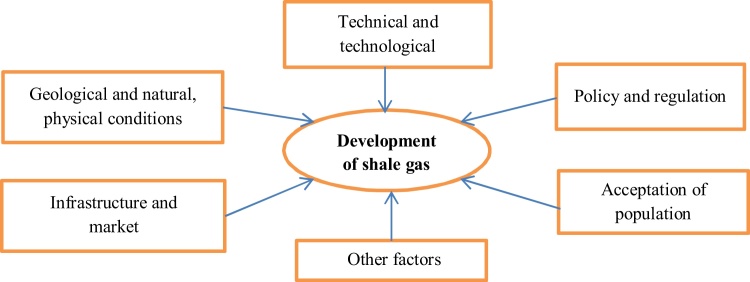
Key conditions influencing the development of shale gas.

The revolution in the US shale gas has triggered a worldwide search for shale gas on other continents, in other countries. However, the conditions for developing these resources, including geological conditions, techniques and technology, natural gas market, infrastructure and regulations, are different. The mains conditions influencing the development of shale gas is presented in Fig. 3. The success of the revolution of shale gas in the US is the combination of a number of favorable factors. The US experiences are lessons for other countries or regions which desire to develop the shale gas industry. In this content, we will analyze and compare the development conditions of shale gas between Europe, China and the United States to clarify the development potential of shale gas in the former two.

### 3.1 Geological and natural physical conditions

#### 3.1.1 Geological condition

The success of the revolution of shale gas in the US is the result of many reasons, but the most important factor is the geological and natural physical conditions because the basins in the United States are very favorable for shale gas development. In the US, shale gas deposits are extensive and rich in organic materials. In general, shale gas formations in the US are shallower than those in other countries. It is estimated that the average burial depth of shale gas in the US is approximately 800–2600 m (Hu and Xu, 2013). Shale layers are thicker and have no faults. According to geologists, the veins of shale gas deposit in the United States have a thickness of 6–200 m. In the United States, the index of TOC (total organic carbon) of shale gas reservoirs is from 0.5 to 25%. The gas content in a ton of shale gas in the United States is quite high: about 40–330 scf/ton (standard cubic feet/ton) (NETL, 2013).

In terms of geography and tectonics, geological conditions of shale gas deposits in China are extremely different from those in the United States. The shale gas in China has good potential for development but the geology is not favorable (Gao, 2012; Stevens et al., 2013). Geological nature of shale gas reserve in China is scatted and deep (Wang and Hefley, 2016). In addition, these shale gas deposits are famous for their rough terrain surrounding the area and transportation difficulty. According to research from EIA in 2013, the most common depth of shale gas deposits in China is between 1700 m and 5500 m (see Table 5), even more than 6000 m as basin Sichuan, and they are structurally more complex with numerous faults (Chou, 2013), while the most popular depth in the US is between 800 and 2600 m. This makes the technological challenges of accessing it in China even greater, and the risks of pollution is higher (Gunningham, 2014). The total organic carbon content in the shale gas in China tends to be lower than that in the US, the TOC index for shale gas in China is from 1.1% to 6.6%. On the other hand, many of the major deposits are rich in clay. These factors make the fracturing process more difficult and also lower productivity. Therefore, to develop shale gas in China, we need more advanced technology; however, the cost of extraction technologies is higher than that in the US (Hu and Xu, 2013; Andrews-Speed and Len, 2015). These shale deposits also contain high concentrations of toxic gases or much more non-hydrocarbon gases. In particular, shale gas formations in southern China contain high levels of hydrogen sulfide. For instance, the concentration of hydrogen sulfide in the Sichuan Weiyuan block is from 0.8% to 1.4%, whereas the northern of Sichuan block reaches 15% (Yu, 2015).

**Table 5:** The geological characteristics of major shale gas plays in the US, Europe and China.

Country/Deposit	Area (square miles)	Depth (ft)	Thickness (ft)	TOC %	Gas content (Scf/ton)
US					
Marcellus	10,4067	4000–8500	50–200	3–12	60–100
Antrim	2400	600–2200	70–120	1–20	40–100
Fayetteville	5853	1000–7000	20–200	4–9.8	60–220
Barnett	5000	6500–8500	100–600	4.5	300–350
Haynesville	9320	10,500–13,500	200–300	0.5–4	100–330

China					Concentration (Bcf/square miles)
Sichuan	74500	9700–13200	251–400	3.0–4.0	109.8–162.6
Yangtze Platform	611,000	11,500–13,200	275-400	3.0–3.2	99.4–147.1
Tarim	234,200	10,790–14,620	160–240	2–3	1.6–85
Songliao	108,000	3300–8200	500	4–5	45

Europe					
Poland	4980–19,700	6000–16,000	182–451	3–3.9	27.4–181.1
UK	3470–10,200	4000–13,000	149–410	3	14.5–117.3
France	17,800–61,000	4000–16,400	83–160	2–9	8.4–61.3
Germany	10,000	3300–16,400	75–90	4.5–8	5.5–56.5
Scandinavia	90,000	3300–15,000	200	7.5	76.8–110.5

Actually, the number of shale gas drilling in Europe is not enough, geological information is therefore weak and uncertain. In comparison with the US, the geological conditions in Europe are more complicated, although faulting appears less prevalent than in other parts of Europe. European shale gas basins tend to be smaller, tectonically more complex, and geological units seem to be more compartmentalized. Furthermore, shale gas tends to be deeper and more pressurized. The quality of the shale is different with generally more clay content in Europe (Gény, 2010). Most European deposits have been identified in geological strata located at comparable depth. According to the figures in Table 5, the majority of resources would be found between 3000 and 4500 m in France, between 3000 and 3900 m in Poland, between 3500 and 4500 m in Germany (EIA, 2013b). The results of initial exploration work – particularly in Poland – have confirmed the complex geology; rock mass is less porous and difficult to fracture. Hence, the development of shale gas in Europe will encounter many challenges.

#### 3.1.2 Population density

The topographic and demographic conditions have exacerbated the difficulties of accessing the land for large-scale drilling, transportation, installation of heavy equipment essential for the development of shale gas (Gunningham, 2014; “Shale Gas”, 2012; Gao, 2012). In the US, the population density is low, the water is available and the topography is relatively flat. These conditions are therefore favorable to the extraction and have significant impact on the operating cost of shale gas in the United States. Accessing land surfaces is one of the greatest challenges that shale gas operators will face in Europe and in China, together with production costs higher than those in the US.

With the population close to 1.4 billion people, China has the highest population density in the world, 145 people/km^2^ compared to 34 people/km^2^ in the United States (see Table 6). Most Chinese shale gas resources are also often located in mountainous terrain, rocky, desert or located in areas where population densities are high. In Europe, although the average population density is not too high, about 32 people per km^2^, many important shale gas deposits in Europe are in highly urbanized areas, in particular Northern Europe such as the Netherlands and northern Germany.

**Table 6:** The population density in the world (persons/km^2^).

Country/Region	2010	2011	2012	2013	2014
***North America***					16
United States	34	34	34	35	34
Canada	4	4	4	4	4
***Europe***					32
France	119	119	120	120	117
Germany	235	235	231	231	232
Poland	125	126	126	126	118
***Asia***					136
China	142	143	144	145	145
India	405	411	416	421	386

High population density means that there are numerous buildings and infrastructure scattered all across regions, and safety zones around these regions also occupy spaces. These will generate a lot of obstacles for operators who want to access the land to develop shale gas. The two real underlying challenges are securing acceptance by local communities and regulations on the location of drilling, fracking operations and safety. The high population density makes the cost of drilling in Europe and China more expensive than that in the United States. Therefore, land access in relation with high population density in Europe and China is a matter of policy, regulation has more restrictive provisions on drilling locations, and safety is a top priority. Thus, access to land is a big challenge in the process of developing shale gas in Europe and China.

#### 3.1.3 Water condition

The hydraulic fracturing process in the development of shale gas consumes a huge amount of water. Therefore, the water condition is an important factor to develop shale gas. In China, water is an urgent problem because China is a country with relatively limited water resources. The available volume of water per person is extremely low (see Table 7), and water resources are unevenly distributed in China – see Fig. 5 (Farah and Tremolada, 2013). Unfortunately, many areas of shale gas reserves in China are facing water supply problems. Most major basins containing Chinese shale gas are located in arid or semi-arid, with rainfall less than 800 mm per year, and many of these basins are densely populated with high pressure to ensure the water supply (Yu, 2015).

**Table 7:** Comparisons of water resources between countries.

Country	Precipitation (Million m^3^)	Internal flow (Million m^3^)	Inflow of surface and ground water (Million m^3^)	Renewable freshwater resources (Million m^3^)	Renewable freshwater resources per capita (m^3^)
United States	6,440,000	2,460,000	18,000	2,478,000	7951
China	6,172,800	2,840,500	21,400	2,861,900	2140
France	485,686	175,293	11,000	186,293	3003
Germany	307,000	117,000	75,000	188,000	2285
Poland	193,100	54,800	8,300	63,100	1656
UK	275,029	157,875	6,405	164,280	2683

**Fig. 4:**
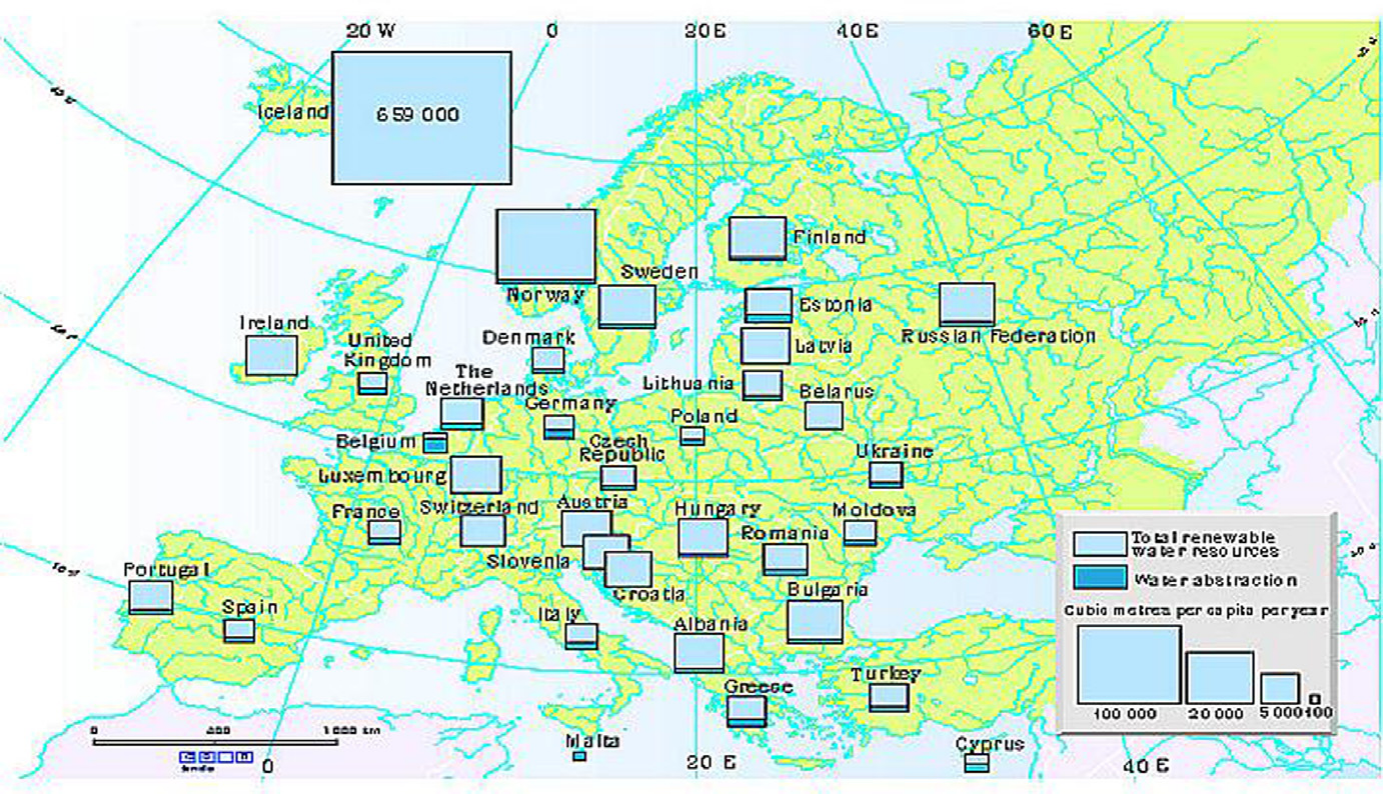
Water Resource in Europe. Source: European Environment Agency (2008).

On the continental scale, Europe seems to have abundant renewable freshwater resources. However, these water resources are unevenly distributed, both between and within countries. In the areas where the population density is high, the inequity is even more striking. Unfortunately, in countries with high potential of developing shale gas such as Poland, the United Kingdom, France, water supply seems to be a potential problem (see Fig. 4 and Table 7).

**Fig. 5:**
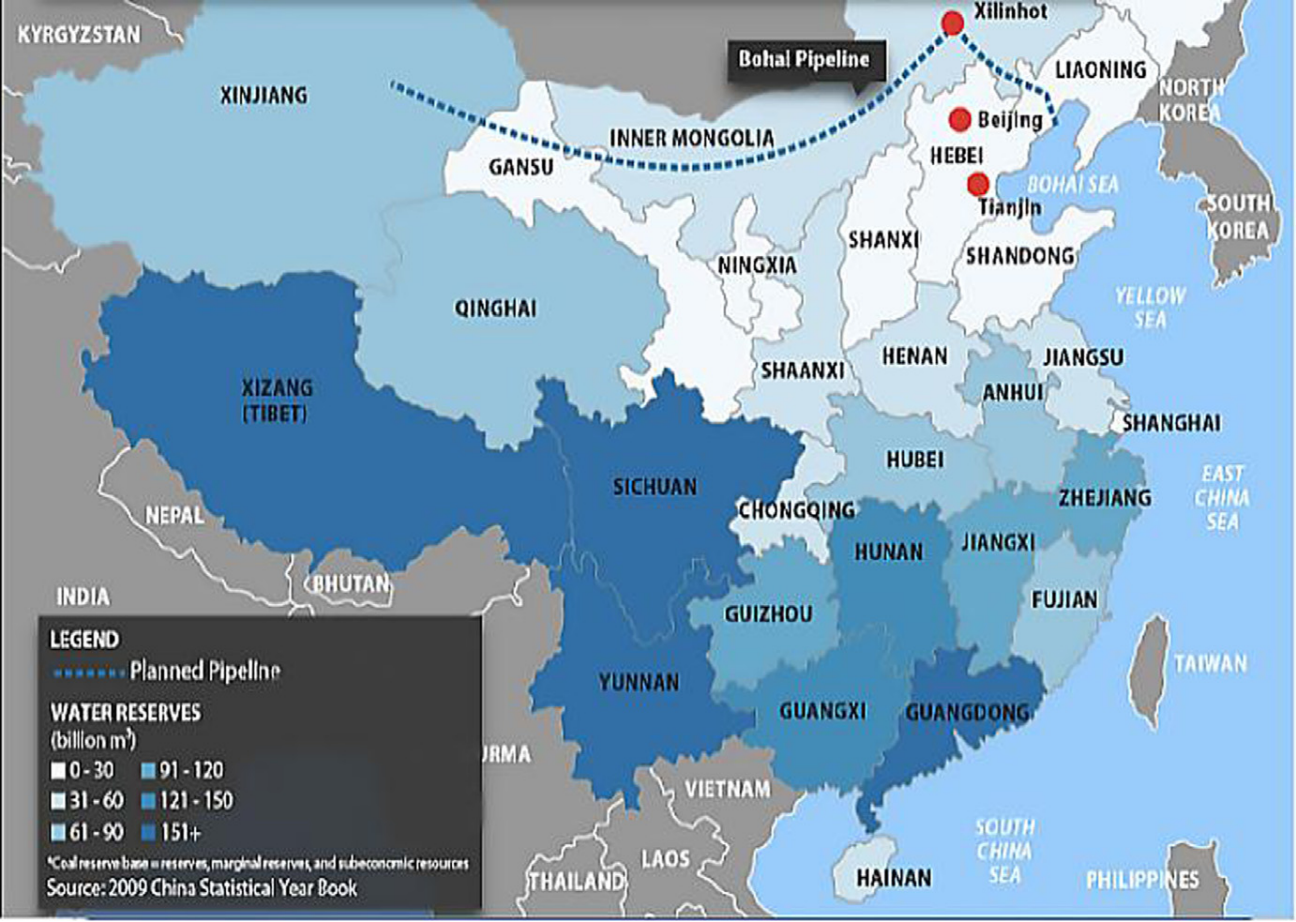
Water Reserves in China. Source: China Statistical Yearbook (2009).

The severity of the problem also depends on the water competition from other economic sectors, especially agriculture, local communities nearby of the basins. A corollary of the issue is the cost of water supply. For example, according to the study of Gény, the price per unit volume of water in Europe is 10 times more expensive than that in the US, with an estimated average cost of 3.4 euros/m^3^ versus 0.4 euros/m^3^ (Gény, 2010). This is a fundamental element that will increase extraction and operating costs of shale gas. Hence, the water shortage is perhaps the greatest natural constraint for the development of shale gas in China and Europe.

### 3.2 Technique and technology condition

The second condition strongly influencing the development of shale gas is technique and technology, especially horizontal drilling and hydraulic fracturing. They play an important role in increasing production and lowering operating costs (Wanniarachchi et al., 2015; Li et al., 2016). In the early 1980s, the US government has initiated research and development programs on the techniques and technologies in the field of unconventional gas. Hence, Americans have mastered the advanced and modern technologies in this field, and the companies also have a lot of experience. In addition, the United States has many service companies, which have a lot of experience and have mastered the technology and techniques with suitable equipment and professional staff, and competitive prices. As a result, the United States has more advantages to develop shale gas compared to other countries in the world.

China's biggest challenge involves technologies, particularly those of horizontal drilling and hydraulic fracturing (Nakano, 2012; Gunningham, 2014). The country lacks necessary personnel and equipment to exploit its shale gas reserves on a large scale. The national oil and gas companies of China lack advanced technology, skills, experience and supply chains to support the rapid and efficient exploitation of shale gas (Hu and Xu, 2013; Ming et al., 2014; Andrews-Speed and Len, 2015).

China has a strong ambition to rapidly develop shale gas, so the Chinese government enforces programs and policies to encourage the development of technologies in this field. There are bilateral cooperation programs for shale gas between the Chinese government and government of the US. In the development plan of shale gas in China for the period of 2011–2015, the Chinese government has promised to increase its investment in research and development of shale gas technologies. To promote such a development, special research funds have been set up by various Chinese government departments (Yu, 2015). Chinese companies have also acquired assets abroad for learning technologies in the field of shale gas. They can access technologies and expertise through direct partnerships with international oil companies (Gunningham, 2014). However, the development of shale gas in China is still in the primary stage, the main technologies such as horizontal drilling and hydraulic fracturing are still at the research stage (Xingang et al., 2013). Therefore, China is coping with a shortage of equipment, expert staff and experience to develop large-scale shale gas.

Europe is in the initial stage of shale gas, although there are major oil companies such as BP, Statoil, Total that have a lot of experience and advanced technologies, most of these companies operate in the field of conventional hydrocarbons and are mainly offshore. Consequently, they do not know many techniques and technologies in the field of shale gas. According to experts, the lack of oilfield service sector capacity, suitable equipment, skilled labor and competition within the sector itself have been highlighted as potential bottlenecks preventing a faster development of shale gas in Europe (Gény, 2010; Stevens, 2012; Spencer et al., 2014). Therefore, the technology factor is an obstacle for shale gas in the old continent.

Shale gas production is an intensive industrial activity, with a large amount of drilling required to achieve and maintain significant production. We must note that the availability of drilling equipment in the US is much higher than that in Europe and China. It can be seen in the US case, where the country averaged 1087 active natural gas drilling rigs per year between 2005 and 2012 (Barbier, 2014). There are approximately 1900 active drilling rigs on the American territory in 2014. Despite the rapid decrease in the amount of drilling platforms to the United States in 2015 because of the fall in oil and gas prices, the number of drilling platforms is still high at about 1000 (see Table 8). On the contrary, the quantity of drilling rigs in Europe and China is very limited.

**Table 8:** Amount of drilling rigs in the world.

Country/Zone	2010	2011	2012	2013	2014	2015	2016
US	1541	1875	1919	1761	1862	977	510
Europe	94	118	119	135	145	117	96
Asia Pacific	269	256	241	246	254	220	187

According to the Strategic Research Center of China Ministry of Land and Resource, to achieve the objectives of the 12th five-year plan of shale gas production in 2020, China will need about 150–250 platforms to implement the drilling plan, which is about 20,000 wells for 10 years. Similarly, in order to achieve the number of drilling rigs respecting the production plan, Europe should strengthen its capacity of 30–40 rigs by year (Gracceva, 2012). However, at present, the principal China drilling rigs are off-shore; there are not many lands rigs in China. In Europe, in 2013, there are about 100 drilling rigs, but there are only a few (over 30 rigs) for natural gas among them, an insignificant fraction of these platforms can be used to drill shale gas wells (Spencer et al., 2014). For example, in 2011, out of 15 drilling rigs available in Poland, only 5 drilling rigs were suitable to shale gas extraction. In both Europe and China, there are just a few manufacturers of land platforms, and they lack the capability and expertise to produce advanced equipment required to develop shale gas.

Furthermore, geological conditions in China and Europe are more complex while the water resources are limited, so the technology used in the United States may be inappropriate for the development of shale gas in China and Europe. Both require more advanced technologies and equipment (Xingang et al., 2013). The companies of China and Europe do not only lack the advanced technology, skills, and experience but they also are in a shortage of management skills in shale gas projects.

### 3.3 Political condition

#### 3.3.1 Opinion and policy in general

The United States has formalized political approaches and been encouraging the development of shale gas. It has established regulations to protect the environment during shale gas development. The regulatory systems are variable and disseminated, including applicable federal, state and local laws and regulations. The US success with shale gas relies on the result of the combination of indulgent federal enforcement, diversified state laws and regulations of local governments. The transparent regulatory regime featuring specific boundaries in federal and state regulations helped investors realize the risks and encouraged them to vigorously invest in new shale basins, which in turn assisted smaller energy firms to refine their technology in hydraulic fracking (Farah and Tremolada, 2013). It has even helped to reinforce public confidence in the field of shale gas (Krupnick et al., 2014). A series of federal laws governs most environmental aspects of shale gas development, for example: the Clean Water Act, The Safe Drinking Water Act, The Clean Air Act, The National Environmental Policy Act (DOE, 2011). The support of the federal government has catalyzed the initiatives of gas operators.

In China, the government has identified shale gas as one of the exceedingly important resources in the strategy of energy development in the future which aims to increase energy self-sufficiency, improve energy structure, and ensure energy safety. The Chinese government has established the program to achieve their ambition for developing shale gas. To stimulate the extraction of shale gas, the Chinese government has promulgated a series of policies in order to promote shale gas exploitation. This political system has four main aspects: industrial planning, support of research and development, tax concessions and subsidies, and innovation in the management mechanisms (Hu and Xu, 2013; Yu, 2015). The concrete objectives of shale gas development policies were clearly expressed in the 12th Five-Year Plan of China. For example, the Chinese government will forward investment and set up funds to support research and evaluation of shale gas, increase its support for innovation and improvement of the technology of shale gas, improving the shale gas infrastructure, study and introduce subsidies and tax credits to accelerate the development of the shale gas industry (Hu and Xu, 2013).

The first observation is that EU environmental laws and regulations will have further impact on shale gas than their energy counterparts, and the impact is direct. Environmental regulations in Europe are much stricter. In particular, using chemical additives in the fracturing fluids, the transportation and storage of flow-back mud from well fracturing, or the drilling of wells within proximity to water reservoirs or inhabitations would completely be extremely difficult or outright forbidden under the current European environmental legislation, both at the Union and at the member state level (Gény, 2010). In addition, the existing regulations in most European countries today are applicable for the exploration and production of conventional oil and gas, but do not take into account the specificities of shale gas. This can lead to legal uncertainty vis-à-vis operational challenges in the shale gas sector. Alongside the inadequate regulatory system on shale gas, laws and regulations available in this sector are not homogeneous and even contradictory in Europe. Furthermore, the opinion and policy about the development shale gas in Europe are very different between the member countries. Some countries have allowed the allocation of shale gas exploration permits such as Denmark, Poland, Romania and the United Kingdom; some countries are in consideration of such authorizations like Germany, Hungary, Portugal, Spain; and there are countries which have banned hydraulic fracturing in their territory such as France, Bulgaria and Czech Republic.

#### 3.3.2 The policy of using the land to develop shale gas

Unlike most of the countries in the world where the government is the absolute owner of all hydrocarbons in the underground, in the US, the private ownership of land is also the owner the underground resources. This law allows landowners to rent or sell their land directly to producers. The owners receive profits from the activities of the producer. Thus, producers may have access to land and resources with less difficulty (Stevens, 2012; Lozano Maya, 2013). They could lease large tracts of land at low prices, and these leases became more valuable as the cost of decreasing extracting gas (Wang and Krupnick, 2013). This foundation has been a major cause of the rapid shale gas development in the US. Moreover, the economic retribution perceived by landowners fosters less social opposition.

Access to the resource is one of the most important issues for shale gas producers in Europe. In most European countries, the underground resources like shale gas are owned by the state. Therefore the local population has no economic incentive for the development, only nuisance (Sylvie Cornot-Gandolphe, 2014). The landowners may not be willing to permit a company to work on their land if the access is not compensated by a financial incentive. The argument runs that mineral right regimes in European countries pose greater challenges for drilling because surface owners are not entitled to royalties or ‘signing bonuses’ and hence have little incentive to support shale gas development (Gracceva, 2012). In addition, because of high levels of population density, the surfaces of concessions in Europe are small (approximately 2.6 km^2^ on average according Gény, 2010), the scale and use of drilling in the area of shale gas will be constrained by space and land access, and the programs of work are very strict.

In China, the land and the mineral resources are owned by the state. The Ministry of Land and Resources will supervise all shale gas acreages. While shale gas is recognized as an independent mineral resource, the shale gas deposits are usually buried under the ground, which can lead to an overlap with other minerals such as coal, oil. In practice, shale gas blocks under exploration or development largely coincide with areas where the Chinese National Oil Companies (NOCs) hold rights on oil and conventional gas (Chou, 2013). Although the Chinese government has committed that shale gas developers take priority when applying for a land use permit, these overlaps will create many difficulties for other companies who want to enter the shale gas industry in China.

#### 3.3.3 Fiscal policy

Financial conditions to exploit shale gas, especially fiscal incentives, also played a key role in the US to promote the exploitation of shale gas. The federal government's fiscal policies have encouraged investment in this sector, in particular by the policies of incentive pricing and the tax credit. Section 107 of the *Natural Gas Policy Act* provided for incentive pricing for shale gas and other forms of unconventional natural gas (Wang and Krupnick, 2013). Notably, the support of federal tax credit provided by the so-called Section 29 played a great role for developing. This tax credit has stimulated the development of unconventional gas. It has increased the financial returns, so it reduces the investing risk in the resources of unconventional gas, as a result, it stimulates investment in the activities of development and improvement of the technology (Wang and Krupnick, 2013).

In Europe, besides the contrasting positions on hydraulic fracturing, the differences in public policies implemented in terms of fiscal incentives for people affected by exploration, exploitation activities and resource-accessing companies are also worth noting. Most fiscal regimes validated in European countries are applied in the sector of conventional oil and gas. Similarly, these tax regimes vary between countries. Except the English and Polish cases, there is no specific taxation on shale gas in Europe. The Polish government presented and submitted for public consultation in 2013 some estimated legislative changes to the country’s tax and regulatory framework for minerals and hydrocarbons. Polish royalty’s rate for shale gas is 1.5% vis-à-vis 3% to conventional gas. The legislation also provides a 20% in tax depreciation rate for wells and drilling and production platforms that are more aggressive than existing corporate income tax treatment (Nülle, 2015). The British government has also announced a new tax regime for the exploration of shale gas. The taxation of revenue from shale gas would be 30%, against 62% for the production of conventional oil and gas (Sylvie Cornot-Gandolphe, 2014).

In China, the government has introduced many incentives to accelerate the development of shale gas, including subsidies and reduction or waiver of the related fees or taxes for the energy companies that engage in investments in shale gas industry. In fact, to promote the exploration and development of shale gas, in 2012, China's Ministry of Finance published a notice about the incentive policy for development and use of shale gas. The policy indicates that the central government has allocated special funds to support shale gas development and use: the subsidy standard for shale gas production companies is 0.4 yuan per cubic meter from 2012 until 2015 (Hu and Xu, 2013). It will be adjusted according to the development of the shale gas industry.

Outside the shale gas subsidy policy, the Chinese government is studying other fiscal policies. The government will reduce the burden of mineral resources compensation fees and user fees for mining rights for shale gas exploration companies (Hu and Xu, 2013; Yu, 2015). In addition, the government will study and implement policies incentive through the tax on resources such as exemption under exploration, mining royalties, VAT, income tax and other taxes in the future. Finally, any imported shale gas exploration and development equipment that is for self-use and cannot be produced in China may be applied for an exemption from customs/duties by the importer (Nakano, 2012; Hu and Xu, 2013; Yu, 2015). These policies will encourage and support the shale gas exploration and exploitation. In fact, thanks to these policies, shale gas production in China has continued to increase in recent years. According to the National Bureau of Statistics, the total production of shale gas in China was about 6.5 Bcm, reaching the target of the 12th Five-year plan for shale gas.

### 3.4 The conditions of infrastructure and market

#### 3.4.1 Infrastructure condition

In order to develop shale gas, we need sufficient infrastructure systems, especially pipeline network. The United States has owned the natural gas pipeline network, compressor stations, storage facilities which were very highly integrated transmission and distribution grids. The natural gas pipeline grid comprises more than 210 pipeline systems, over 1400 compressor stations that maintain pressure on the natural gas pipeline network and assure continuously forward movement of supplies. There are about 500,000 km of interstate and intra-state transmission pipelines with over 11,000 delivery points, 5000 receipt points and 1400 interconnection points that are provided for transferring natural gas throughout the United States. It also counts 400 underground natural gas storage facilities and several regasification and liquefaction plants (EIA, 2010). Therefore, the gas market is excessively developed; it makes the transport and use more easily, and renders the cost cheaper.

Economic efficiency of shale gas development is highly dependent on the distance between the production area and the end customers. But most Chinese reserves of shale gas, such as the Sichuan Basin in the southwest, the Tarim basin in the northwest, are located in areas far from the existing pipeline network and away from the market consumption. Unlike the United States, China does not have an extensive network of pipelines nationwide. Although China has intensified its pipeline development in recent years, the construction of the pipeline has not kept pace with the rapid growth in demand. In the 12th five-year plan, China also plans to build 44,000 km of new natural gas pipelines (Chou, 2013). The total length of China’s pipeline network – approximately 50,000 km – accounts for only one tenth of that of the United States (see Table 9), while large diameter trunk lines only account for 15,000 km. As a result, the insufficient gas transmission has significantly constrained the growth of shale gas in China.

**Table 9:** Comparison of natural gas pipeline system among US, Europe and China.

	US	Europe	China
Total length of pipeline (km)	500,000	200,000	50,000
Pipeline density (km/1000 km^2^)	50	30	5

The total length of pipeline systems in Europe is approximately 200,000 km. There are roughly 30 km of transmission pipelines in 1000 km^2^ in Europe, compared with 53 km in the USA (EIA, 2011; Gracceva, 2012). Although, the gas infrastructure has significantly expanded over the past recently decades in Europe, several new supply projects are currently under study or under construction. However, there are no pipeline projects related to the development of shale gas in Europe. In addition, the infrastructure system of natural gas between member countries in Europe is difference. The major internal pipelines in Europe are not homogeneous, as there is little connection between the Western pipeline network and the Eastern infrastructure. Moreover, shale gas deposits in Europe are far removed from the main pipeline networks.

Another obstacle is the access to transport capacity. In the US, the numerous interconnecting infrastructures allow access to a fully liberalized market and encourage competition among gas operators, whereas access to natural gas transmission pipeline capacity in Europe and China was controlled and dominated by large companies of national public service or national oil companies. Therefore, it created many challenges for operators in the shale gas sector.

#### 3.4.2 Market structure

Market structure is an important factor when we consider the possibilities of a development of shale gas on a large scale. It is likely the most underappreciated factor that positively benefited growth in shale gas production in the United States (Medlock, 2012). The US has a fully liberalized market for natural gas but the reforms to the EU’s internal gas market are still ongoing and very different from the China’s gas market. The success of the US relies on the efforts of thousands of small and medium sized enterprises, and a competitive market is advantageous for the development of shale gas. These enterprises have played the role as pioneers in the exploration of shale gas and led to the large increases in shale gas production (Bauquis Pierre-René, 2014). According to statistics of Boiteux et al. in 2010, there were nearly 8000 gas producers, 200 private companies and gas transportation in 1400–1500 local distribution companies, sometimes private and sometimes public.

Unlike the United States, the gas market in China is a monopole market. China does not have thousands of independent oil and gas companies that compete with others. The oil and gas industry in China is a monopoly industry for a long time through the NOCs. Most exploration and production licenses for oil and gas in China are divided between NOCs, including the rights of the most attractive areas for shale gas. There is no competition in the energy sector either upstream or downstream, which created monopolies in markets and prices of administrative frameworks. The China’s service companies in oil and gas are associated with major NOCs and the NOCs tend to use such companies to provide services. Hence, these companies dominate the internal market for services and have strong bargaining power vis-à-vis local governments and contractors. These national oil companies such as CNPC, Sinopec, CNOOC dominate and control most pipelines. Consequently, the barriers to entry in the field of shale gas, especially for private and foreign companies are very large.

The European case is the same as that of China. The gas market in Europe is the same market oligopoly. The liberalization process in the gas sector is ongoing. The European gas industry is dominated by a few large players and therefore a highly concentrated market. In particular, players in the upstream and midstream of the gas industry in Europe are still the major companies or some national companies. Furthermore, the sector of gas services in Europe is less developed than that in the USA and the competition is not significant. In Europe, the liberalized gas market is not yet fully defined in particular in terms of conditions for an access to the transmission network.

The gas price in the United States is now linked to the physical balance of supply and demand on the spot market, and the price mechanism is the gas-on-gas; while traditional gas prices in European continental were determined by long-term contracts using formulae embracing oil prices and the cost of primarily oil-based products (Asche et al., 2012). Although, gas hub liquidity in Europe has grown over the recent years, like NBP in the UK, TTF in the Netherland; the gas price has been moving away from oil-indexation towards gas-on-gas competition, but in reality it has not been universal across Europe, the oil-indexation is still dominating in Europe.

The China’s gas pricing system is a compromise between market prices and administered prices. In the past, China's governments have held the price of gas at a low level to protect consumers. The gas price varies according to consumers, depending on the level of the offer by the wholesale or retail, onshore or offshore, etc (Siliverstovs et al., 2005). Government interventions under the monopoly of power leading to the current prices do not reflect the scarcity of resources and demand on the market. The price control system in the current natural gas in China prevents the development of the natural gas market (Nakano, 2012). Although there were reforms of gas pricing in China recently, the large gap between domestic prices and international prices of natural gas will constrain the development both of the gas market and of shale gas in China.

### 3.5 Population acceptance

One of the key factors of the exploration and production of shale gas is the societal acceptance of hydraulic fracturing, the only proven technology to date. There were a lot of controversies related to the environment issues, the environmental concerns have created strong opposition to fracking, not only in the US but also in other regions, other countries. Although some operations in shale gas are beginning to face an increase in local opposition in the United States, the acceptance of development of shale gas is acquired in most regions of the nation. Because the country has a long history of onshore oil and gas activity, the population density is low and the resources of water are abundant. Besides, the US regime of landholder ownership of subsoil mineral rights has been an important factor in public acceptance of shale gas, as exploitation means royalties to landholders.

In the European case, this condition is likely to present the major challenge to develop shale gas. The opposition to the exploitation of shale gas is rapidly increasing in Europe, especially the environmental concerns that have created a strong opposition to fracking. In addition, environmental regulations are stringent; Europe is densely populated and highly urbanized. Thus large-scale shale gas operations will impinge on local communities. The governments and industries in Europe are often submitted to strong local opposition. It led to several moratoria or fracking bans in European countries. In late 2012, the results of the public consultation on the development of shale gas in Europe showed that nearly 60% of the European citizens are opposed to shale gas production (European Commission DG Environment, 2013; EY, 2013). Therefore, the challenges in relation to public opinion that shale gas developers have to face seem to be even greater in Europe.

In China, until now there were not many reports of resistance from local communities on shale gas operations, although the structure of politic and society of China has created significant differences in the development of shale gas between China and the United States. The government owns the mining and pilot projects, so individual landowners are less likely to benefit from drilling activity and associated shale gas activities (Davies and Tomsen, 2013). However, the challenges facing China are formidable since shale gas development is accompanied by potentially severe environmental damage and risks to human health. If shale gas operations cause unacceptable harm to water, environment, land or human life, especially in densely populated regions, local communities could begin to resist development plans of shale gas in China.

## 4 Conclusion

The development of shale gas in the United States results from the combination of many favorable factors. These are geological and natural conditions and the mastery of advanced technologies. The policies are appropriate, synchronous, detailed and transparent to encourage the development of shale gas. These policies aim at protecting the environment, as well as creating public confidence and acceptance. Moreover, the shale gas development requires a developed and liberalized market, providing a competition with an appropriate price mechanism. It also requires a developed infrastructure, including networks of pipelines for transporting and distributing natural gas which contribute to the reduction of the cost.

The boom of shale gas in the US has become a powerful catalyst for other countries that have the potential of shale gas resources. Recognizing the importance of natural gas as a source of transition energy, several governments have strong incentives to replicate US success in their countries. The experiences of the United States in the exploration, development, production of shale gas could be useful for other countries to develop their own shale gas industry.

Analyzing and comparing the conditions which help to develop shale gas in the countries/zones that have potential shale gas resources, and the desire of shale gas development will show the potential development of these resources in those countries/zones. Relying on the basis of the factors that contribute to the success of shale gas in the US, we have analyzed and evaluated the conditions for the development of shale gas in Europe and in China. All the analyses and evaluations are summarized in Table 10. There are many important differences among the US conditions and those of Europe and China such as the diversity of geology, technology level, infrastructure, population density; policies and regulation systems, etc.

**Table 10:** Comparisons of conditions for the shale gas development among the US, China and Europe.

Content		The US	Europe	China
Geology and physical conditions	Resources	- Many drilling rigs, a lot of information - High reserves, fourth in the world	- Lack of information, uncertainty - Approximately 10% of world reserves of shale gas	- Lack of information, uncertainty - The largest estimated reserves in the world
	Geology	Favorable	More complex	More complex
	Water source	- Available - 7951 m^3^/person	- Low in countries where there is shale gas potential - Ex: Poland 1656 m^3^/person	- Limited, water is an urgent problem - 2140 m^3^/person
	Population	Low density: 34 persons/km^2^	High density in countries that have potential of shale gas (Poland 118/km^2^)	High density: 145/km^2^
	History and status	- More than 80 years - Large-scale production, commercial utilization	- At the end of the 2000s - In the infancy stage	- At the end of the 2000s - Testing and drilling wells
Technical and technological conditions		- Mastery of technology - Many service companies, equipment, drilling rigs and experienced staff	- Lack of service companies, suitable equipment and skilled labor. - Limitation of drilling rigs	- Ongoing learning - Lack of service companies, experienced staff - More advanced technologies in need - Very few drilling rigs
Political Conditions	General policy	- Strongly support - System of policy is complete, synchronic, transparent and disclosure	- Depend on countries - Insufficient system of policy, regulations of environment is very strict	- Strongly support - Lack of political system
	Policy of the land use	Private property	State property	State property
	Fiscal policy	Fiscal policies have encouraged investment	Except English and Polish, there are no specific fiscal policies	There are fiscal policies to support
Conditions of infrastructure		- Very high integration in transport and distribution network 500000 km, 50 km/1000 km^2^	- The infrastructure is very difference in the member countries, the pipeline is far from deposits 200000 km, 30 km/1000 km^2^	- Insufficient transmission pipeline 50000 km, 5 km/1000 km^2^
Market structure		- Fully liberalized market - Many small private companies with competition	- Oligopoly market and ongoing liberalization process - Dominated by a few major companies or some national companies, lack of competition	- Monopole market and ongoing transition from monopoly to competition - The NOCs dominate
Regime of price		- Linked to the physical balance of supply and demand on the spot market - Mechanism: gas-on-gas	- Hybrid, gas price has been moving away from oil-indexation towards gas-on-gas competition - The oil-indexation is still dominant	- Very different: between domestic and international, and among customers - Market prices and administered prices
Social acceptance		Accepted in most of the US	Around 60% of the European population are opposed	No reports of resistance

Our analysis has indicated that no other region possesses these favorable conditions and that is probably the reason why a success in shale gas development has so far been only observed in the US. This also indicates that China and countries in Europe will face significant challenges that will take time to resolve. The uncertain potential of resources, imperfect political system, environmental problems, and backward infrastructure are the main threats and weaknesses that China and Europe have to face in the exploration and development process. Although China is developing shale gas and started its production of shale gas in Sichuan, some countries in Europe such as Poland, the UK are exploring and drilling shale gas, but in the current conditions, when oil prices dropped sharply in the world, the energy policy and climate change policies have had many changes that will strongly impact the development of shale gas in the world. The US’s shale revolution appears impossible to replicate in both China and Europe.

## Declarations

### Author contribution statement

Minh Thong Le: Conceived and designed the experiments; Performed the experiments; Analyzed and interpreted the data; Contributed reagents, materials, analysis tools or data; Wrote the paper.

### Competing interest statement

The authors declare no conflict of interest.

### Funding statement

This research did not receive any specific grant from funding agencies in the public, commercial, or not-for-profit sectors.

### Additional information

No additional information is available for this paper.
